# Coflex Interspinous Stabilization with Decompression for Lumbar Spinal Stenosis: An Average 14-Year Follow-Up

**DOI:** 10.3390/jcm14082856

**Published:** 2025-04-21

**Authors:** Juneyoung Heo, Ji-Hoon Baek, Ji Hyun Kim, Jae Chil Chang, Hyung-ki Park, Su Chan Lee

**Affiliations:** 1 Joint & Arthritis Research, Department of Neurosurgery, Himchan Hospital, Seoul 07999, Republic of Korea; juneyoungheo@gmail.com; 2 Joint & Arthritis Research, Department of Orthopaedic Surgery, Himchan Hospital, Seoul 07999, Republic of Korea; jihoon011@naver.com (J.-H.B.); tttis5555@gmail.com (J.H.K.); 3 Department of Neurosurgery, Soonchunhyang University Seoul Hospital, Seoul 04401, Republic of Korea; j7chang@schmc.ac.kr (J.C.C.); phk007@schmc.ac.kr (H.-k.P.)

**Keywords:** spinal stenosis, instability, Coflex, interspinous, degenerative

## Abstract

**Background:** This study aimed to evaluate the long-term clinical usefulness and radiologic changes around the Coflex device following decompression with Coflex insertion for degenerative lumbar spinal stenosis (DLSS), with an average follow-up of 14 years. **Methods:** This retrospective study included 147 patients who underwent decompression and Coflex insertion for single-level DLSS at a single institution between January 2007 and December 2010. Patients with spinal stenosis unresponsive to 3 months of conservative treatment were treated surgically. The mean follow-up duration was 173.9 ± 23.7 (range, 119–214) months. **Results:** The mean visual analog scale score decreased from 8.22 ± 1.06 preoperatively to 2.08 ± 1.58 postoperatively. Intervertebral disc height and foramen height at the Coflex insertion site decreased by 5.3% and 2.0%, respectively, after surgery. The reoperation rate at the operated site was 25% (*n* = 37). A significantly higher reoperation rate was observed in patients with translational instability (odds ratio [OR], 7.77; 95% confidence interval [CI], 2.453–24.658; *p* < 0.01) and angular instability (OR, 1.59; 95% CI, 0.492–5.133; *p* < 0.001). Eight patients underwent reoperation due to rapid progression of instability within 2 years of Coflex insertion; thereafter, a similar cumulative incidence rate was consistently observed. The adjacent-segment reoperation rate was 10.8% (*n* = 16). **Conclusions:** The Coflex interspinous device helps preserve disc and foramen height but is associated with a high reoperation rate, particularly in patients with spinal instability. Therefore, careful patient selection is crucial when considering its use.

## 1. Introduction

Degenerative lumbar spinal stenosis (DLSS) is a common condition in older adults, and its prevalence is steadily increasing due to the rapidly aging population [1]. DLSS involves narrowing of the spinal canal or neural foramina, which compresses the nerves and surrounding tissues, resulting in back pain and neurogenic intermittent claudication [2,3]. Degenerative changes in the spine, such as reduced intervertebral disc height (IDH) and facet joint hypertrophy [4], can contribute to segmental instability [5]. Furthermore, decompression of neural elements—which involves the removal of bone, ligament, and disc material—can lead to instability of the posterior column [6]. For many years, spinal fusion with instrumentation has been the standard surgical treatment to stabilize the affected segment [4].

However, one of the major limitations of spinal fusion is adjacent-segment degeneration (ASD), which is so common that its incidence has been reported to reach up to 89% within 5 years after surgery [7]. Other complications, such as pseudoarthrosis and implant failure, are also relatively frequent [8,9]. These issues arise because although spinal fusion stabilizes the treated segment, it increases mechanical stress on adjacent segments, leading to significant kinematic alterations [10,11]. To address these concerns, spine specialists have, since the 2000s, explored alternative methods that preserve physiologic load transfer to reduce pain and prevent ASD [12,13,14,15,16]. One such method is the use of the Coflex device. The Coflex dynamic stabilization system (Paradigm Spine, LLC, New York City, NY, USA) is a U-shaped, compressible titanium alloy implant classified as an interspinous process device (IPD). It is designed for insertion into the interspinous space and has been widely adopted since the 2000s.

IPDs, including the Coflex, have demonstrated promising results in biomechanical and early clinical studies [17,18,19,20]. However, subsequent research has raised concerns about their limited long-term clinical efficacy and implant-related complications. The main issues associated with IPDs include high reoperation rates, increased complication rates, elevated costs, and excessive loading on spinous processes—commonly referred to as the “sandwich phenomenon” [1,21,22,23]. Additionally, some studies suggest that IPDs accelerate the degenerative cascade [24]. Notably, these findings are based on studies with follow-up periods of <8 years, and there has been limited investigation on the underlying causes or predisposing factors of these complications.

In this study, we analyzed patients who underwent decompression with Coflex insertion for DLSS and were followed for an average of 14 years. We evaluated the long-term clinical outcomes and radiologic changes associated with the Coflex device, identified predisposing factors influencing these outcomes, and assessed its impact on lumbar spine degeneration.

## 2. Materials and Methods

This retrospective study included 147 patients who underwent decompression and Coflex insertion surgery for single-segment DLSS at a single institution between January 2007 and December 2010. The study was approved by the Institutional Review Board of Himchan Hospital, and patient confidentiality was strictly maintained.

All patients underwent detailed history taking, as well as physical and neurological examinations, to assess for radiating pain and neurogenic claudication of the lower extremities, which are hallmark symptoms of DLSS. Lumbar roentgenography, computed tomography (CT), and magnetic resonance imaging (MRI) were performed to identify lesions corresponding to the patients’ symptoms. Prior to surgery, all patients received conservative treatment, including nonsteroidal anti-inflammatory drugs, physical therapy, and epidural steroid injections, for >3 months. Decompression and Coflex insertion surgery were performed only when there was no clinical improvement following these conservative measures.

### 2.1. Patient Selection

The inclusion criteria were as follows: (1) patients with lumbar spinal stenosis of Schizas grade B or higher at a single level (L3–L4, L4–L5, or L5–S1) on MRI [25], presenting with severe radiating leg pain with or without neurogenic claudication, whose symptoms did not improve after >3 months of conservative treatment, and who subsequently underwent decompression and Coflex insertion surgery, and (2) patients without spondylolisthesis or spinal stenosis in the adjacent segments.

The exclusion criteria were as follows: (1) history of prior lumbar spine surgery before decompression and Coflex insertion; (2) spondylolisthesis of Meyerding grade II or higher at the affected level [26]; (3) Coflex insertion involving more than two spinal segments; (4) significant scoliosis with a Cobb angle of >25°; (5) rapidly progressing motor weakness or neurological symptoms consistent with cauda equina syndrome; and (6) presence of concomitant conditions that could significantly affect surgical outcomes, such as spinal fractures, infections, deformities, tumors, ankylosing spondylitis, or pregnancy.

### 2.2. Radiologic Analysis

The degree of pain was measured using a visual analog scale (VAS). The IDH and intervertebral foramen height (IFH) were measured, and vertebral slippage was assessed using lumbar roentgenography. Radiographs were obtained in two positions: anteroposterior and lateral views in the standing position and lateral views in dynamic (flexion and extension) positions. IDH was defined as the vertical height at the midpoint between adjacent vertebrae on the lateral standing X-ray. IFH was defined as the distance between the lower edge of the upper vertebral pedicle and the upper edge of the lower vertebral pedicle on the same image. IDH and IFH were measured at the operated segment as well as at the adjacent segments above and below. Translational and angular instability were defined according to a previous systematic review [27]. Translational instability was defined as a difference of ≥3 mm in vertebral body translation between flexion and extension views on standing lateral X-rays, whereas angular instability was defined as intervertebral mobility of ≥10° relative to the adjacent level. Follow-up assessments were conducted at 1 month, 3 months, and 1 year postoperatively and then every 2 years thereafter. Clinical outcomes were evaluated using VAS and X-ray imaging during these follow-up visits. If acute symptoms developed before the scheduled follow-up, additional imaging such as CT or MRI and laboratory tests were performed as needed, and appropriate management was provided. For patients unable to visit the hospital in person, follow-up was conducted via telephone, and X-rays taken at local facilities were reviewed. If the patient had died, follow-up was discontinued.

### 2.3. Reoperation and Surgical Complications

“Same-segment reoperation” was defined as a surgical procedure performed at the same level as the initial decompression and Coflex insertion for lumbar spinal stenosis or lumbar herniated intervertebral disc, when severe lower extremity radiating pain, with or without back pain, did not improve after >3 months of conservative treatment. “Adjacent-segment surgery” was defined as the same condition occurring at a segment adjacent to the originally treated level, requiring surgical intervention. Spinal fusion was performed in cases involving the same or adjacent segment when instability was present, central and foraminal stenosis coexisted, or bilateral foraminal stenosis was observed due to a significant reduction in IDH. For other types of lesions, decompression alone or discectomy was performed.

Following Coflex surgery, if infection or inflammation developed around the Coflex device, or if implant-related adverse effects such as abnormal fluid accumulation or a foreign body sensation occurred and device removal was deemed necessary, the Coflex device was surgically removed. In cases where instability needed to be corrected or an increase in IDH was required due to symptoms such as vertebral slippage, reduced IDH, or foraminal stenosis at the same level postoperatively, posterior lumbar interbody fusion was performed after removal of the Coflex device. If no symptoms were present and no significant changes in the condition of the Coflex device—such as breakage, compression, or displacement—were observed on X-ray imaging, no additional intervention was undertaken, and the patient continued with regular follow-up monitoring.

Statistical analyses were performed using the paired *t*-test, analysis of variance, and Pearson correlation coefficient. All *p*-values were two-sided, and a *p*-value of <0.05 was considered statistically significant. Data analysis was conducted using IBM SPSS Statistics for Windows, version 27 (IBM Corp., Armonk, NY, USA).

### 2.4. Surgical Procedure

After the induction of general anesthesia, the patient was placed in the prone position for surgery. A standard midline incision was made at the surgical site under fluoroscopic guidance. The supraspinous ligament and spinous process were exposed by subperiosteal dissection of the paraspinal muscles on both sides. The interspinous ligament was removed while preserving the integrity of the supraspinous ligament. To decompress the nerve root until it could move freely, partial removal of the upper and lower laminae was performed, along with careful undercutting facetectomy and ligamentum flavum removal. A Coflex device of appropriate size was then positioned at the site of the removed interspinous ligament and securely fixed under fluoroscopic guidance. The procedure was then completed.

## 3. Results

The mean age at the time of surgery was 60.9 ± 6.26 (range, 51–78.8) years, and the study included 46 male and 101 female patients (Table 1). The mean height, weight, and body mass index (BMI) of the patients were 157.9 ± 8.4 cm, 62.2 ± 9.05 kg, and 24.9 ± 3.1 kg/m^2^, respectively. The most common level for Coflex insertion was L4–L5 (121 patients, 82%), followed by L3–L4 (14 patients, 9%) and L5–S1 (12 patients, 8.2%). In terms of comorbidities, 58 patients (39.5%) had hypertension, and 16 (10.9%) had diabetes. The mean follow-up duration was 173.9 ± 23.7 (range, 119–214) months.

Most patients who underwent decompression and Coflex insertion surgery experienced postoperative symptom improvement. The mean preoperative VAS score was 8.22 ± 1.06, which decreased to 2.05 ± 0.99 at 1 month postoperatively. The VAS score remained relatively stable throughout the follow-up period, with a mean score of 2.08 ± 1.58 at the final follow-up.

### 3.1. IDH and IFH at the Coflex Insertion Site and Adjacent Levels

During the follow-up period, the IDH at the level of Coflex insertion decreased by 0.83 ± 2.7 mm (5.3% ± 0.23%), and the IFH decreased by 0.78 ± 3.6 mm (2% ± 0.25%) (Table 2). At the adjacent levels, the IDH decreased by an average of 0.41 mm (3.3% ± 0.43%) above and 0.24 mm (1.1% ± 0.31%) below the insertion site. The IFH at the levels above and below decreased by 1.86 mm (4.6% ± 0.26%) and 1.14 mm (7.6% ± 0.26%), respectively. Although IDH and IFH values decreased at the Coflex insertion site and at both adjacent levels, the differences were not statistically significant (*p* = 0.179–0.677).

### 3.2. Same-Segment Reoperation

A total of 37 patients (25%) underwent reoperation at the same segment, 12 of whom had documented instability prior to the initial Coflex surgery (Table 3 and Table 4). Among these, 10 patients had translational instability, 5 had angular instability, and 3 had both angular and translational instability. Specifically, five patients had only translational instability, whereas three had both types. Eight patients (five with translational instability and three with both angular and translational instability) underwent Coflex device removal followed by spinal fusion within 2 years after the initial surgery due to persistent or worsening symptoms. One patient underwent device removal due to infection 1 month postoperatively, and another had the device removed because of a foreign body sensation and severe back pain attributed to the Coflex implant. The remaining 20 patients underwent laminectomy or discectomy—without fusion—for spinal stenosis or lumbar herniated intervertebral disc at the same site, at an average of 102.8 ± 39.7 (range, 59–198) months postoperatively. In this study, demographic factors (sex, age, height, weight, BMI, hypertension, and diabetes mellitus), surgical level, and preoperative VAS score—excluding the presence of instability—were not significantly associated with same-segment reoperation, adjacent-segment surgery, or other surgical complications.

### 3.3. Adjacent-Segment Surgery

A total of 16 patients (10.8%) underwent surgery at the adjacent segment, 7 of whom had instability at the original Coflex insertion site (Table 3 and Table 4). Among these, five had angular instability, five had translational instability, and three had both angular and translational instability. The average time to adjacent-segment surgery was 117 ± 50.5 (range, 50.7–188.9) months. The incidence and timing of ASD in patients with instability were not significantly different from those in patients without instability. Of the 16 patients with ASD, 14 had spinal stenosis or lumbar herniated intervertebral disc without instability at the adjacent segment and underwent laminectomy or discectomy alone. The remaining two patients developed spondylolisthesis with foraminal stenosis at the adjacent level and required fusion surgery.

### 3.4. Other Postoperative Complications

Changes in the shape or position of the Coflex device were observed in 17 patients (Table 4). In five patients, fractures occurred at the upper or lower fixation site, although no positional changes were noted afterward. Eight patients had fractures in the middle of the device, five had fractures at one fixation site, and two showed device bending. The average time to detection of a Coflex shape change was 86.8 ± 47.9 (range, 2–155) months. Additionally, in two patients, the device had migrated 5 mm posteriorly from its original position, as observed on X-ray images taken 3 and 5 years after insertion; however, neither patient reported foreign body sensation or back pain. Among those with changes in Coflex shape or position, two underwent reoperation at the same segment: One had surgery 35 months after a fracture was observed at the upper fixation site, and another underwent surgery 30 months after a mid-device fracture was noted. One patient had surgery at the adjacent segment and later underwent an additional procedure 43 months after a fracture was detected at the inferior fixation site.

## 4. Discussion

### 4.1. IDH and IFH Preservation

In this study, changes in IDH and IFH appeared to be primarily related to aging. Given the gradual decrease observed over the follow-up period, it is difficult to attribute degenerative changes at the same or adjacent segments solely to the Coflex device—except in cases involving spondylolisthesis or instability. Conversely, the relatively minor reductions in IDH (5%) and IFH (2%) over an average follow-up of 14 years may clinically support findings from previous biomechanical studies [28,29], which suggests that Coflex reduces intradiscal pressure and helps slow disc degeneration. Furthermore, changes in IDH and IFH were not significantly associated with reoperation at the same segment.

### 4.2. Same-Segment Reoperation

**Figure 1:**
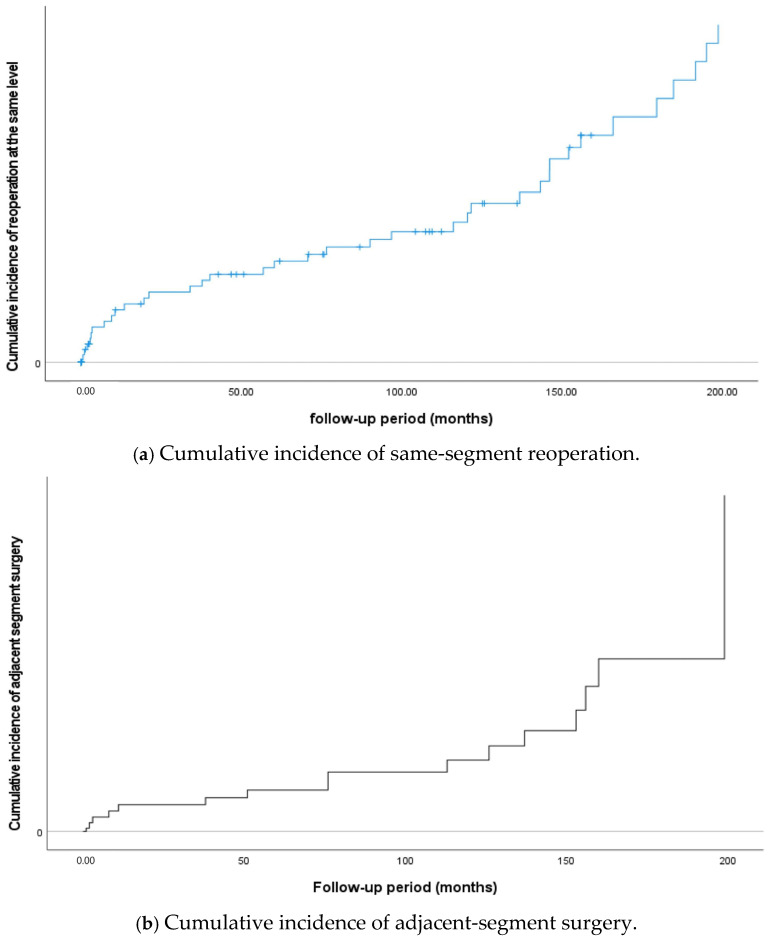

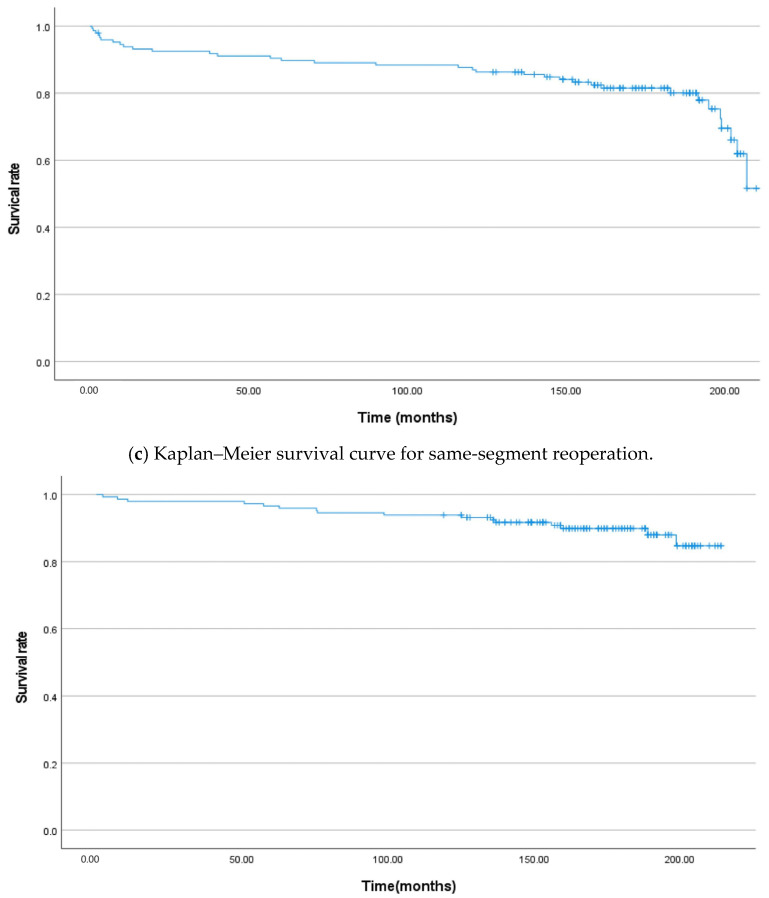
Cumulative incidence of reoperation and Kaplan–Meier survival curves for same-segment and adjacent-segment surgeries during the follow-up period.

Eight patients underwent reoperation at the same segment due to rapid progression of instability within 2 years following Coflex insertion, and similar cumulative incidence rates were observed consistently thereafter (Figure 1a,c). In a prospective controlled study with a 2-year follow-up, Richter (2014) reported no reoperations [21], whereas subsequent studies with follow-up periods exceeding 7 years have reported same-segment reoperation rates of approximately 10–15% [18,20,29]. In the present study, the reoperation rate at the same segment was 25% (*n* = 37). Given that the follow-up period in this study was more than twice as long as those in earlier reports, the results may still be considered generally consistent with the existing literature. Compared to other interspinous devices, which have shown reoperation rates exceeding 25% [1,30], Coflex may offer a relatively lower reoperation rate when long-term follow-up is taken into account. Nevertheless, considering the intended purpose of Coflex—to provide a motion-preserving alternative to fusion—the findings of this study are not entirely favorable. Several systematic reviews have indicated that interspinous devices are associated with higher reoperation rates compared to both fusion and decompression alone [1,22,30,31]. The long-term outcomes of Coflex observed in this study are similar to those reported in other studies of IPDs [19,22,27,30]. Although the sustained improvement in clinical symptoms and the preservation of IDH and IFH represent notable advantages, the high reoperation rate and occurrence of surgical complications remain key disadvantages of IPDs, including Coflex.

In this study, the only factor significantly associated with an increased rate of reoperation at the same segment was preoperative instability. Among the patients who underwent reoperation at the same level, eight with documented preoperative instability required fusion surgery due to the rapid progression of instability within 2 years. This finding suggests that, unlike fusion surgery, Coflex insertion does not adequately address segmental instability. Notably, the reoperation rate was significantly higher in patients with translational instability (odds ratio [OR] 7.77, 95% confidence interval [CI] 2.453–24.658, *p* < 0.01) and also higher in those with angular instability compared to those without (OR 1.59, 95% CI 0.492–5.133, *p* < 0.001). Previous studies have shown that Coflex reduces the range of motion in patients with angular instability [29,32,33], and a similar effect was observed in the dynamic X-ray images taken during the follow-up period. However, in the present study, the impact of Coflex on reducing the range of motion was minimal. Based on these findings, we believe that Coflex insertion should be avoided in cases where preoperative instability is present.

Several studies on interspinous devices have identified a high reoperation rate in patients with degenerative spondylolisthesis as a major limitation [34,35,36]. One study even concluded that degenerative spondylolisthesis should be considered a contraindication for their use [23]. Davis et al. [37] reported that Coflex produced outcomes comparable to fusion in cases of low-grade spondylolisthesis; however, the reoperation rate was still 14.1% during a minimum 2-year follow-up. The high reoperation rates and rapid clinical deterioration observed in patients with instability in the present study further support these previous findings. As noted in the Introduction, Coflex is intended to provide spinal stabilization as a motion-preserving alternative to fusion. However, its effectiveness appears to be significantly limited in cases where adequate stabilization cannot be achieved.

### 4.3. Adjacent-Segment Surgery

In this study, the adjacent-segment surgery rate was 10.8% (*n* = 16), with the incidence increasing over time. Notably, the rate remained relatively stable for up to 10 years following Coflex insertion but increased more rapidly thereafter (Figure 1b,d). In previous studies with 7–8 years of follow-up, the rate of ASD-related reoperation was reported to be 6–7% or lower [18,20,29]. Given that the incidence of ASD tends to increase with longer follow-up durations, the rate observed in this study is considered acceptable. Taken together with findings from previous studies, our results suggest that Coflex insertion offers an advantage over fusion surgery in minimizing ASD.

### 4.4. Other Surgical Complications

Among the 17 patients who experienced changes in the shape or position of the Coflex device, none reported worsening symptoms. Specifically, the middle portion of the device fractured in eight patients, indicating potential loss of mechanical function; however, there were no notable differences in VAS scores or clinical outcomes compared to other patients. Three patients underwent surgery at the same or adjacent segment after changes in the Coflex were observed. However, due to the small number of cases and the long interval (30–43 months) between the detection of the change and the subsequent surgery, a direct causal relationship with the device alteration is uncertain.

### 4.5. Clinical Implications of This Study

This study is significant in that it presents the longest follow-up period reported for the use of interspinous devices. However, it included only patients who underwent a single type of surgery and did not compare outcomes with other surgical approaches, such as decompression alone or fusion, limiting the ability to draw comparative conclusions. As a retrospective study, it is subject to inherent biases. Data were collected solely from past medical charts and radiologic records, which may introduce inaccuracies or incomplete information. Furthermore, due to the nature of retrospective research, not all confounding variables could be controlled, and unrecognized factors may have influenced the results. The accuracy of VAS scores may also be affected by chronic pain or the presence of other comorbid conditions. Additionally, the nonrandomized design and single-institution setting limit the generalizability of the findings.

## 5. Conclusions

The Coflex interspinous device offers the advantage of preserving IDH and IFH. However, its use is associated with a relatively high reoperation rate and suboptimal outcomes, particularly in patients with preoperative instability. Given these findings, the widespread use of Coflex appears to be limited, and its application should be carefully selected. In particular, Coflex insertion is not recommended in cases where preoperative instability is present.

## Figures and Tables

**Table 1:** Demographic characteristics and procedural details of 147 patients who underwent Coflex interspinous stabilization with decompression for lumbar spinal stenosis.

Sex (%)	
Male	46 (31.3%)
Female	101 (68.7%)
Age (years)	60.9 ± 6.29 (51–78.8)
Male	59.99 ± 6.5
Female	61.36 ± 6.15
Height (cm)	157.9 ± 8.44
Weight (kg)	62.2 ± 9.05
BMI (kg/m^2^)	25.0 ± 3.10
Male	24.3 ± 2.59
Female	25.3 ± 3.28
Hypertension (%)	
Male	58 (39.5%)
Female	89 (60.5%)
DM (%)	
Male	16 (10.9%)
Female	131 (89.1%)
Level (%)	
L3/4	13 (8.8%)
L4/5	121 (82.3%)
L5/S1	13 (8.8%)
VAS score	
Before surgery	8.30 ± 1.10
One month after surgery	2.03 ± 1.01
Most recent follow-up	2.08 ± 1.58
Follow-up period (months)	173.9 ± 23.7 (range, 119–214)

Data are presented as mean ± SD unless otherwise indicated. BMI, body mass index; DM, diabetes mellitus; SD, standard deviation; VAS, visual analog scale.

**Table 2:** Changes in intervertebral disc height and intervertebral foramen height at the operated and adjacent segments during the follow-up period after decompression and Coflex insertion.

	Preoperative Height (mm)	Decrease at Last Follow-Up (mm)	Percentage Decrease (%)
IDH at the same segment	13.5069 ± 3.32	0.8322 ± 2.75	5.35%
IFH at the same segment	15.0007 ± 4.07	0.7895 ± 3.61	2.86%
IDH at the upper segment	14.2037 ± 2.92	0.4181 ± 7.43	3.37%
IFH at the upper segment	18.4980 ± 9.16	1.8671 ± 9.58	4.69%
IDH at the lower segment	13.4499 ± 3.50	0.2406 ± 2.94	1.12%
IFH at the lower segment	12.6710 ± 3.75	1.1476 ± 3.53	7.6%

Data are presented as mean ± SD unless otherwise indicated. IDH, intervertebral disc height; IFH, intervertebral foramen height; SD, standard deviation.

**Table 3:** Relationship between instability and reoperation or adjacent-segment surgery according to the presence or absence of instability.

(a) Translational Instability							
		Same-Segment Reoperation			Adjacent-Segment Surgery		Total
		Performed	Performed Within 2 Years	Not Performed	Performed	Not Performed	
Translational instability	Yes	10	8	5	5	10	15
	No	27		105	11	121	132
Total		37		110	16	131	147
**(b) Angular Instability**							
		**Same-Segment Reoperation**			**Adjacent-Segment Surgery**		**Total**
		**Performed**	**Performed Within** **2 Years**	**Not ** **Performed**	**Performed**	**Not ** **Performed**	
Angular instability	Yes	5	0	15	3	17	20
	No	22		105	11	116	127
Total		27		120	14	133	147
**(c) Combined Translational and Angular Instability**							
		**Same-Segment Reoperation**			**Adjacent-Segment Surgery**		**Total**
		**Performed**	**Performed Within** **2 Years**	**Not ** **Performed**	**Performed**	**Not ** **Performed**	
Combined instability	Yes	5	3	3	5	3	8
	No	22		117	11	128	139
Total		27		120	16	131	147

**Table 4:** Same-segment reoperation, adjacent-segment surgery, and other postoperative complications following decompression and Coflex insertion surgery.

	N	Subgroup	N
Same-segment reoperation	37 (25%)	Coflex removal and fusion due to instability within 2 years	8 (5.4%)
		Coflex removal and fusion due to instability after 2 years	4 (2.7%)
		Laminectomy or discectomy	20 (13.6%)
		Coflex removal due to infection	1 (0.6%)
		Coflex removal due to foreign body sensation	1 (0.6%)
Adjacent-segment surgery	16 (10.8%)	Laminectomy or discectomy	14 (9.5%)
		Fusion due to instability	2 (1.3%)
Change in Coflex shape or position	17 (11.5%)	Implant broken	13 (8.8%)
		Implant bending	2 (1.3%)
		Position shifted	2 (1.3%)

## Data Availability

The data presented in this study are available upon reasonable request from the corresponding author.

## Abbreviations

The following abbreviations are used in this manuscript:

DLSS	Degenerative lumbar spinal stenosis
IPD	Interspinous process device
VAS	Visual analog scale
IDH	Intervertebral disc height
IFH	Intervertebral foramen height
CT	Computed tomography
MRI	Magnetic resonance imaging
ASD	Adjacent-segment degeneration
OR	Odds ratio
CI	Confidence interval
BMI	Body mass index
